# Bidirectional and Opposite Effects of Naïve Mesenchymal Stem Cells on Tumor Growth and Progression

**DOI:** 10.15171/apb.2019.063

**Published:** 2019-10-24

**Authors:** Faramarz Rahmatizadeh, Shiva Gholizadeh-Ghaleh Aziz, Khodadad Khodadadi, Maryam Lale Ataei, Esmaeil Ebrahimie, Jafar Soleimani Rad, Maryam Pashaiasl

**Affiliations:** ^1^Department of Molecular Medicine, Faculty of Advanced Medical Science, Tabriz University of Medical Science, Tabriz, Iran.; ^2^Student Research Committee, Tabriz University of Medical Sciences, Tabriz, Iran.; ^3^Drug Applied Research Center, Tabriz University of Medical Sciences, Tabriz, Iran.; ^4^Department of Biochemistry, Faculty of Medicine, Urmia University of Medical Sciences, Urmia, Iran.; ^5^Murdoch Children’s Research Institute, Royal Children’s Hospital, The University of Melbourne, Melbourne, Australia.; ^6^Department of Anatomical Sciences, Faculty of Medicine, Tabriz University of Medical Sciences, Tabriz, Iran.; ^7^Adelaide Medical School, University of Adelaide, Adelaide, Australia.; ^8^School of Animal and Veterinary Sciences, University of Adelaide, Adelaide, Australia.; ^9^Department of Reproductive Biology, Faculty of Advanced Medical Science, Tabriz University of Medical Science, Tabriz, Iran.; ^10^Women’s Reproductive Health Research Center, Tabriz University of Medical Sciences, Tabriz, Iran.

**Keywords:** Mesenchymal stem cells, Dual effects, Bidirectional effects, Anti-tumor, Cell-cell interactions, Secretory factors, Tumor progression

## Abstract

Cancer has long been considered as a heterogeneous population of uncontrolled proliferation of different transformed cell types. The recent findings concerning tumorigeneses have highlighted the fact that tumors can progress through tight relationships among tumor cells, cellular, and non-cellular components which are present within tumor tissues. In recent years, studies have shown that mesenchymal stem cells (MSCs) are essential components of non-tumor cells within the tumor tissues that can strongly affect tumor development. Several forms of MSCs have been identified within tumor stroma. Naïve (innate) mesenchymal stem cells (N-MSCs) derived from different sources are mostly recruited into the tumor stroma. N-MSCs exert dual and divergent effects on tumor growth through different conditions and factors such as toll-like receptor priming (TLR-priming), which is the primary underlying causes of opposite effects. Moreover, MSCs also have the contrary effects by various molecular mechanisms relying on direct cellto- cell connections and indirect communications through the autocrine, paracrine routes, and tumor microenvironment (TME).

Overall, cell-based therapies will hold great promise to provide novel anticancer treatments. However, the application of intact MSCs in cancer treatment can theoretically cause adverse clinical outcomes. It is essential that to extensively analysis the effective factors and conditions in which underlying mechanisms are adopted by MSCs when encounter with cancer.

The aim is to review the cellular and molecular mechanisms underlying the dual effects of MSCs followed by the importance of polarization of MSCs through priming of TLRs.

## Introduction

Mesenchymal stem cells (MSCs) are multipotent, self-renewing, and heterogeneous population of mesenchymal progenitor cells which exhibit at least three main characteristics based upon the minimal definition criteria recommended to define human MSCs by The International Society for Cell and Gene Therapy (ISCT, Europe), approved in 2006 and outlined as below:

First, MSCs have a physical property of plastic adherence when they are maintained under defined culture conditions.

Second, MSCs exhibit specific cell surface markers like CD73, CD90, CD105, and negative expression of CD14, CD45, CD34, and human leukocyte antigen (HLA)- DR.

Third, MSCs also have an intrinsic ability to differentiate into chondroblasts, adipocytes, and osteoblastsin vitro conditions.^1,2^

So far, MSCs have been derived and purified from many different sources such as embryonic/fetal and non- embryonic sources of connective tissues.^3-5^

Indeed, MSCs obtained from different sources have shown many different behavioral and biological characteristics that might be more closely related to their origins.^6-9^ The most obvious biological differences among MSCs are listed below:

They may present different cell surface marker profiles, genetic diversities, broad differentiation potential, and may show different proliferation rates. MSCs have an inherent tendency to migrate toward inflammatory sites, primary tumor sites and metastatic foci via chemotaxis.^10,11^ Only a minor subpopulation of non-tumor cells within the tumor tissues are MSCs and this has been estimated to be about 0.01-1.1 percent of the total number of cells.^12^ Despite the fact that, almost a small number of MSCsare present in the tumor tissues they have important role in determining the fate of tumor cells.^13,14^ There are three main types of MSCs within tumor stroma including: (1) N- MSCs ornormal MSCs, hereinafter referred to as MSCs, (2) Tumor tissue-educated MSCs or tumor associated MSCs (TA-MSCs), and (3) cancer-associated fibroblasts (CAFs).^15,16^

Wide range of heterogeneity also exists among different tumor tissues. Hence, not only host individuals have unique conditions but also each tumor type has its own unique characteristics^17,18^ that may be altered dynamically at different stages of tumor progression.^19^ The distinct differences among different types of tumors are termed inter- and intra- tumor heterogeneity that have a correlation with genetic and epigenetic modifications in tumor cells.^20^ Taken together, during tumor progression the heterogeneous and innate population of MSCs (which has unique properties and functions) can engraft within tumors, and consequently interact with highly heterogeneous components of tumor microenvironments (TMEs). These components are made of various different combinations of tumor tissues such as bulk tumor cells as well as non-tumor-initiating cells. Therefore, it has been predicted that the different MSCs may exert multiple effects on various tumor types.^21-24^ Moreover, previous experimental results have shown that MSCs have dual effects on tumor growth and progression. Thus, they may exhibit pro- and anti-tumorigenic activities when confronting with cancers through cytokines, chemokines, growth factors, and many different factors indirectly or via direct cell-to-cell connections.^25-32^ Obviously, there are so many variables which may subsequently determine the functional effects of MSCs on tumor development.^33^ For instance, variations that exist in MSCs isolated from different sources, the cross-contamination of MSCs with cancer cell lines,their behavior in a time and concentration dependent manner, the effects of route of MSC administration, the influence of different toll-like receptors (TLRs) on MSCs, and predicting MSC behaviors at different time pointsare more important than other conditions and factors.^33-37^ As a result, the dual and opposite effects of MSCs on tumor progression can restrict the utilization of these cells in the field of cell-based cancer therapies.^38^ In order to overcome the major impediments to identify definitive solutions for cell-based cancer therapies which are more effective, it is necessary to gain in depth the knowledge of the possible underlying molecular mechanisms through which MSCs can exert their opposite effects on tumor cells. Moreover, we have emphasized the importance of a new paradigm which can promote the anti-tumor effects of MSCs via stimulation of TLR4. This concept has indicated that TLR4-priming results in polarization of MSCs into a pro-inflammatory phenotype or MSC 1 which has shown to exhibit anti-tumor properties.^33,39^

### Tumor-promoting effects of MSCs

There is empirical evidence to demonstrate that MSCs pose supportive effects on tumor growth and progression.^40,41^ These cells have been identified that can promote tumor growth and progression in mouse models of gastric cancer, gliomas, colon cancer, subcutaneous breast tumors, prostate cancer, ovarian cancer, head and neck cancer *in vitro.*^23,42-49^ Besides, the supportive effects of MSCs (through using different molecular mechanisms) have an impact on all three major steps of tumorigenesis process which consists of initiation, promotion, and progression.^41^

#### The promotion of tumor growth by modulating immune responses

**Figure 1:**
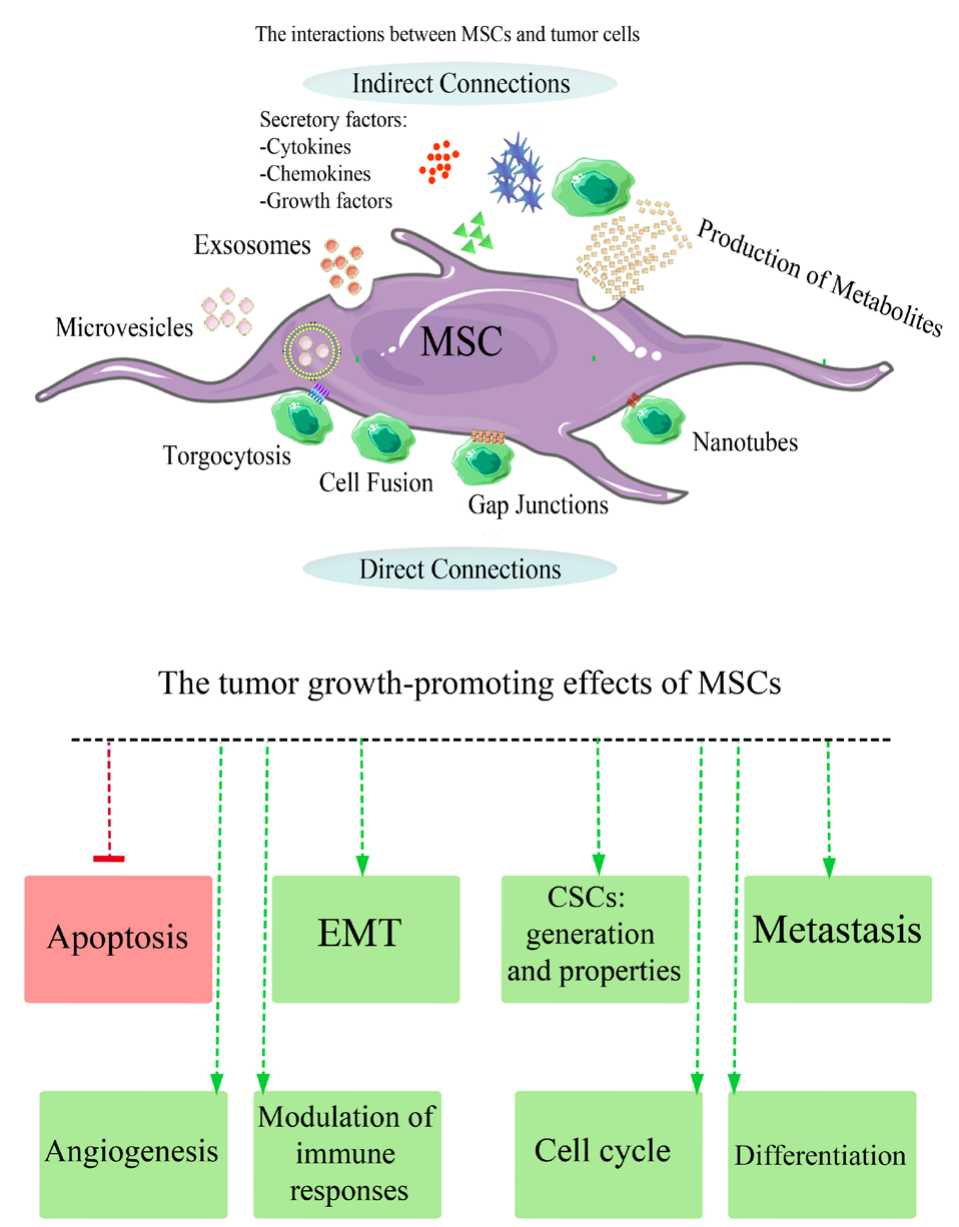
Different types of communication between MSCs and tumor cells and various mechanisms adopted by these cells to promote tumor growth and progression directly and indirectly.
EMT, Epithelial –to- Mesenchymal transition; CSCs, Cancer Stem cells; MSCs, Mesenchymal Stem cells.

MSCs derived from various sources have a profound impact on innate and adaptive immune responses that finally may result in the promotion of tumor progression.^50^ The regulatory effects of MSCs are exerted on immune responses through direct cell-cell communication and indirectly by the release of particular secreted factors such as nitric oxide (NO), transforming growth factor beta (TGF-β), prostaglandin E2 (PGE2), Interleukin (IL)-6, indoleamine-2, 3-dioxygenase (IDO), interleukin (IL)-10, and HLA-G5 (Figure 1).^51-56^

Tumor-associated macrophages (TAMs) are considered as the decisive components of TME that have an influence on immune responses and might be involved in tumor-related inflammation.^57^ TAMs have at least two different states of polarization in the TME including the classically activated M1 macrophage phenotype and alternatively activated M2 macrophage phenotype.^58^ The polarization of TAMs can be derived toward an M1-like phenotype that exerts noticeable pro-inflammatory and anti-tumor activities. On the contrary, when TAMs switch to the M2 phenotype display pro-tumor activities and may be involved in the resolution of tumor inflammation. MSCs can reciprocally modulate the polarization of TAMs toward the M2 phenotype that can promote tumor progression.^59^ The other effects of MSCs on innate immune cells that are associated with the enhancement of tumor progression include reduction in proliferation, differentiation, activation, and functions of natural killer (NK) cells through expression of PGE2, TGF-β1, and IDO.^60^ Also, cytokine-activated MSCs can suppress maturation, migration, and antigen-presentation capacity of monocyte derived dendritic cells.^51,61,62^ MSCs can block degranulation of the mast cells and prevent oxidative burst and apoptosis of human neutrophil.^63^

Additionally, MSCs are able to modulate adaptive immune responses and promote the tumor progression through multiple mechanisms.^64^ These cells have significant effects on T-lymphocytes and the induction of T-cell clonal energy. In this regard, MSCs also inhibit both CD4+ and CD8+ T-lymphocyte proliferation through direct contact. These cells also secrete a large number of different growth factors that result in cell cycle arrest in G1/G0 phase. MSCs not only can reduce the number of CD8+ and CD4+T lymphocytes but also inhibit the production of interferon-alpha (IFN-α), interferon-gamma (IFN-γ), and interleukin (IL)-1 which are secreted by T-cells.^61,65-70^ However, MSCs do not exert their inhibitory effects on CD4 + T-cells which are activated through IFN-γ.^71^ Programmed cell death-1 (PD-1) pathway plays a critical role in regulating T-cell homeostasis and activation. These cells can directly activate the PD-1 pathway via cell-to-cell communication between T-cells and MSCs or indirectly through secretion of PD-1 specific ligands including B7-H1 (PD-L1/CD274) and B7-DC (PD-L2/CD273). The linages are able to suppress CD4^+^ T-cells, downregulate IL-2, induce irreversible hyporesponsive state and cell death.^72^ MSCs also reduce the number of T-cells through expression of Fas ligand (FasL)^73^ (Figure 1) and are capable of suppressing the activation of CTLs and skew the differentiation pattern of naïve CD4+ T-cells (Th0) into T -helper type1 cells (Th1), T-helper type 2 cells (Th2), and T-helper type 17 cells (Th17) that finally result in the production of Th2 cells and enhanced the release of IL-4.^74,75^ In addition, MSCs may help to improve the production of CD8+ or CD4+ regulatory T-cells (Tregs).^52,76^ Taken together, they skew the Th1/Th2 cytokine balance toward Th2 (or an anti-inflammatory state) in tumor stroma.^77,78^ Furthermore, MSCs showed inhibition effects on multiple aspects of B lymphocyte activity including proliferation, functions, chemokine receptor expression patterns, and differentiation into antibody-secreting cells.^79,80^

#### Differentiation and transdifferentiation of MSCs into other cell lineages

The tumor mass consists of a highly diverse and heterogeneous cell population include bulk tumor cells and tumor-associated stromal cells (TASCs)^81^ that plays a significant role in tumor progression.^82^ MSCs have capability to differentiate into various mesenchymal lineage cell lines (Figure 1).^83-85^ For instance, MSCs are one of the sources of CAFs and it has been estimated that approximately less than 20 percent of CAFs may be originated from differentiation of MSCs.^86^ In addition, CAFs are able to support tumor growth and angiogenesis by producing stromal cell-derived factor-1 (SDF-1) and increasing the recruitment of endothelial precursor cells into the tumor site.^87,88^ After differentiation of MSCs into CAFs, the CAFs can enhance tumor progression and metastasis by contributing to remodeling of extracellular matrix (ECM) and inducing epithelial-to-mesenchymal transition (EMT) (Figure 1).^89^

Moreover, MSCs have also been shown to differentiate into other stromal cell lines such as pericytes and endothelial cells. They can rarely undergo neoplastic transformation which in turn may induce angiogenesis and tumor progression.^90^

#### Induction of epithelial–to-mesenchymal transition

EMT process is a biological phenomenon that plays a crucial role in invasion, metastasis as well as acquisition of chemotherapy and apoptosis resistance in tumor cells.^91^ There are highly sophisticated networks of biological signaling pathways involved in EMT process including Notch, Hedgehog, nuclear factor κ-light-chain enhancer of activated B lymphocytes (NF-kB), TGF-β, and Wnt/Wingless (Wg) signaling pathway.^92-95^ Moreover, EMT process can be facilitated by the presence of inflammatory mediators and cells such as pro-inflammatory and inflammatory cytokines or the presence of reactive oxygen species (ROS) that are induced under hypoxic conditions in tumor tissues. In this regard, the existence of hypoxia provides an opportunity to induce the formation of ROS.^96^ MSCs which are recruited into tumor-associated stroma (TAS) can also stimulate ROS formation and have a significant effect on EMT process.^97^ MSCs can facilitate EMT process in tumors through their direct and indirect interaction with tumor cells.^98,99^ For instance, an *in vitro* direct co-culture experiment between colon cancer cells and MSCs has indicated that the crosstalk between MSCs and tumor cells via direct contact results in the overexpression of EMT-related genes such as fibronectin (FN), secreted protein, acidic and rich in cysteine , galectin-1, but these results are not obtained from indirect co-culture conditions.^100^

MSCs as well as TASCs can secrete a variety of paracrine factors such as fibroblast growth factor (FGF), platelet-derived growth factor (PDGF), epidermal growth factor (EGF), hepatocyte growth factor (HGF), and TGF-β that can significantly enhance or stimulate the EMT process (Figure 1).^91,101-103^ One of the most significant signaling pathways that is classically associated with EMT process includes the TGF-β/ mothers against decapentaplegic (SMAD)/lymphoid enhancer-binding factor (LEF)/PDGF axis.^104^

The tumor-associated fibroblast (TAFs)/CAFs as well as myofibroblasts can originate from MSCs and play an important role in inducing and maintaining the inflammatory responses through releasing of pro-inflammatory mediators leading to the activation of EMT process (Figure 1).^81,105^ In addition, CAFs have been shown markedly to exert higher expression of fibroblast growth factor receptor 4 (FGFR4) by which induce EMT process in colorectal cancer cell lines.^106,107^

Interestingly, another possible mechanism which may be involved in EMT process is the spontaneous hybridization between MSCs and tumor cells (Figure 1). Recent investigations have suggested that non-small-cell lung carcinoma (NSCLC) cells after co-culturing with human bone marrow-derived MSCs (hBM-MSCs) are capable of producing hybrid cells *in vitro*. The hybrid cells derived from hybridization between NSCLC and MSCs, exhibit similar biological properties of both EMT and stem cell-like cells.^108,109^

#### Effects of MSCs on cancer stem cells

Various studies have found strong evidence for the existence of cancer stem cells (CSCs).^110^ According to this view, it is widely accepted that CSCs can be isolated from various hematological malignancies and solid tumors.^111,112^ CSCs are rare cells and share many characteristics with MSCs including self-renewal, stemness-related gene expression profiles, the capability of differentiation, the use of common signaling pathways, and the creation of stem cell niches.^113,114^ CSCs are able to differentiate and transdifferentiate into endothelial cells, other types of TASCs, and probably respective tumor types. They also exhibit high metastatic potential and are associated with the chemoradiation resistance. It is suggested that CSCs may be responsible for an increased rate of tumor recurrence.^113,115^

**Figure 2:**
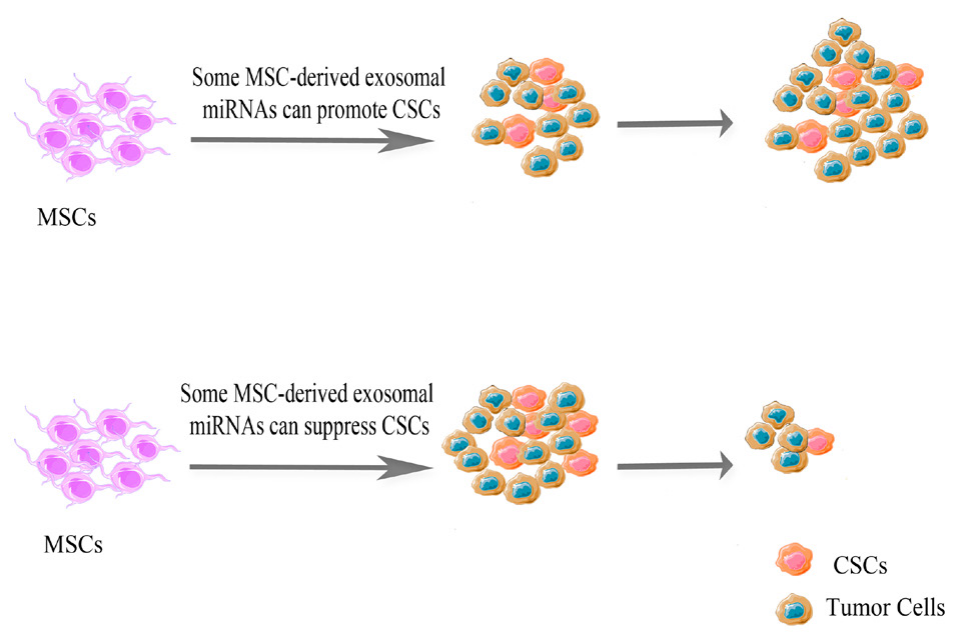
Mesenchymal stem cells play a pivotal role both directly or indirectly in tumor growth and progression, via regulation of cancer stem cell generation and their properties. For instance, MSCs indirectly regulate cancer stem cell niches via mesenchymal stem cell-derived extracellular vesicles (MSC-EVs) and also the release of cytokines such as IL-6, IL-8, IL-12, and gremlin. CSCs, cancer stem cells; MSCs, mesenchymal stem cells.

MSCs have a potent effect on the promotion of expansion and modification of CSCs and by which can enhance their biological functions. In this regard, it has demonstrated that co-culturing MSCs with gastric cancer cells led to an increase in the percentage of CD133-positive cells in the cultures.^99,116-118^ Similarly, it has concluded that after co-culturing MSCs with breast cancer cells, the ALDEFLUOR-positive cells (aldehyde dehydrogenases, ALDHs are markers for cancer stem-like cells in multiple tumor types) increased significantly in culture. They suggested that malignant cell-derived IL-1 resulted in a marked increase in PGE2 secreted by MSCs. The PGE2 and IL-1 were able to secrete synergistically several cytokines by MSCs. The secreted cytokines that was accompanied with PGE2 could promote the activation of beta-catenin (β-catenin) in tumor cells that finally led to CSCs generation.^116,118^ Both TA-MSCs and N-MSCs could create new CSCs by applying the above mentioned procedures.^119^ In addition, MSCs could secrete a wide array of regulatory molecules such as CXCL1, 5, 6, 7, and 8 which are ligands for the chemokine receptor CXCR2. It is noteworthy that, the CXCR2/CXCR2 ligand biological axis plays an important role in supporting the CSCs generation.^116^ Moreover, IL-6, CCL5, bone morphogenetic proteins, and also the expression of chemokine receptors CXCR4 and CXCR7 have also been implicated in the creation of CSCs in various types of cancer.^120,121^ MSCs also express several microRNA (miRNA) profiles which can be involved in the expansion of CSCs^122^ including MiR-302, MiR-21, MiR-106b-25, MiR-181, MiR-495, MiR9/9^*^, MiR-22, MiR-328-3p, MiR-214, MiR-130b, and MiR-199a-3p (Figure 2).^123,124^

#### Stimulation of tumor metastasis

MSCs can promote tumor growth, dormancy, invasion, and their metastatic process through indirect and direct connections.^125-127^ with tumor cells andstromal components of the tumor. The reciprocal interaction between tumor cells and MSCs may induce MSCs to express ligands of CCR2 and CXCR2, and also CCL5.^128^ Among the chemokine/ chemokine receptors that are stimulated by MSCs, the CCL2/CCR2 axis has a crucial role in acquisition of metastatic properties by tumor cells. MSC-derived CCL2 is an important chemokine receptor-ligand that mediates the recruitment of CCR2+ neutrophils into the tumor stroma.^128^ After initial recruitment the interaction between CCR2+ neutrophils and tumor cells leads to a significant increase in metastasis-related gene expression such as CXCR4, CXCR7, matrix metalloproteinase (MMP)-13, MMP-12, IL-6, and TGF-β in tumor cells.^128,129^

Interestingly, tumor cells cannot orient themselves toward metastatic sites.^128^ Indeed, during the metastatic process tumor fragments which consist of the bulk tumor cells, TASCs, and MSCs are held together to form the pre-metastatic clusters. Moreover, the MSCs which are retrieved from the metastatic sites express CAF marker genes including alpha-smooth muscle actin, SDF-1-α, tenascin-C, MMP-9, and MMP-2.^130,131^

#### Induction of anti-apoptotic effects in tumor cells

Emerging evidence indicates that tumor progression is a process that is accompanied by adaptation to anaerobic conditions. Indeed, chronic inflammatory status, hypoxia, acidic pH conditions, and nutrient deprivation have been observed during the tumor development.^132-134^ Under stress conditions MSCs are capable to maintain their primitive characters and functions such as self-renewal capacity and differentiation potentials by the activation of autophagy pathways. MSCs are able to secrete a number of pro-survival and anti-apoptotic factors such as vascular endothelial growth factor (VEGF), insulin-like growth factor (IGF)-2, insulin like growth factor (IGF)-1, PDGF, SDF-1-α, basic fibroblast growth factor (bFGF), HGF, IGF binding protein-2 (IGFBP) -2, stanniocalcin-1(STC-1), and NO (Figure 1).^135-140^ Among them the VEGF family which is known as a survival factor can act by increasing the B-cell lymphoma 2 (BCL-2)/BCL-2 associated X protein (BAX) ratio in tumor cells.^141^ In a similar manner, bFGF can also enhance BCL-2 expression.^142^ Hypoxia induce MSCs to secrete various factors including VEGF, IGF-1, HGF, and bFGF which may enhance tumor cell survival by the activation of angiogenesis and exerting anti-apoptotic effects.^143,144^ On the other hand, an increase in PDGF and TGF-β expression is usually associated with upregulation of VEGF and bFGF genes in tumor cells.^137^ Furthermore, MSC-derived IL-6 exerts chiefly an anti-apoptotic effect on cancer cells and also may enhance tumor resistance to chemotherapy through activation of signal transducers and activators of transcription 3, and upregulation of BCL-2.^145^ In this regard, the evidence obtained from earlier studies that have been carried out on lung cancers (originating from epithelial tissues) suggests that STC-1 can inhibit apoptosis in tumor cells and promote tumor cell survival. The presence of STC-1 in tumor stroma is both necessary and sufficient for the inhibition of apoptosis in tumor cells. MSCs can also prevent apoptosis through the release of NO in a dose-related manner. Therefore, NO can inhibit the apoptosis in tumor cells at a relatively low concentration. Conversely, a high concentration of NO induces apoptosis in tumor cells.^146^ Furthermore, exosomes generated by MSCs can induce tumor progression by inhibiting tumor cell death. It has been established that apoptosis-related miRNAs, which are transferred from MSCs, are critical regulators of tumor cell apoptosis (Figure 1). In this respect, several studies have revealed that miRNAs produced by MSCs are capable of predicting the clinical outcomes in several tumor types such as colorectal cancers, breast cancers, and gastric cancers through inhibition of apoptosis in tumor cells.^147^

#### Induction of angiogenesis and neovascularization in tumors

Tumor angiogenesis and neovascularization are complex multistep processes which occur during tumor progression and can facilitate tumor growth and metastasis.^148^ MSCs because of their potential to secrete wide variety of growth factors, cytokines and chemokines can effectively promote/support angiogenesis through several mechanisms include:

1. MSCs are capable to differentiate into CAFs, smooth muscle cells (SMCs), endothelial cells, and pericytes (nevertheless, transition of MSCs to endothelial cells remains controversial).^149,150^
2. MSCs are able to release a large number of secretory factors to stimulate the tumor angiogenesis in a paracrine manner have been shown markedly to exert higher.
3. MSCs could induce angiogenesis through MSC- derived miRNAs.^152,153^
4. MSCs interact with multiple cell types in TME which can potentially contribute to tumor angiogenesis (Figure 1).^151,154-162^

The new studies have emphasized that MSCs which are located in perivascular niches share several phenotypic and functional characteristics with pericytes.^161^ Besides, accumulating evidence suggests that MSCs can also increase angiogenesis and contribute to vascular remodeling during the formation of tumor vasculature.^163,164^ The other emerging role of MSCs that facilitate tumor angiogenesis and malignant progression is the secretion of cytokines, chemokines, and growth factors by MSCs which are involved in angiogenesis process such as VEGF, FGF-2, HGF, interleukin(IL)-8, TGF-β, MMPs, CXCL8, CXCL2.^161,165,166^

Furthermore, MSCs can also enhance tumor neovascularization through differentiation into CAFs, recruitment of macrophages, and production of extracellular vesicles (Figure 1).^165,167^

Interestingly, empirical evidence has revealed that a particular vessel region which exists in human blood vessels. This vascular mural zone, also referred to as vasculogenic zone, is located at the outer elastic membrane just at the border between tunica adventitia and tunica media which contains a population of vascular wall-resident CD44+ multipotent stem cells of mesenchymal origin (VW-MPSCs). The VW-MPSCs seem to represent the “first line” cells that migrate from their niches to tumor stroma. Besides, the VW-MPSCs are able to differentiate into vascular smooth muscle cells as well as pericytes.^167-169^

The findings indicate that MSCs express a primary receptor also known as the vascular endothelial growth factor receptor 1 (VEGFR1 or Flt-1) on their surface, which is involved in promoting tumor angiogenesis.^170^ Implantation of BM derived MSCs and AT derived MSCs presented controversial outcome in which AT-MSCs stimulate tumor growth and metastatic aggression of cancer while BM-MSCs transplantation presented different effects. Indigenous MSCs may act as supporter of tumor growth after surgical resection through regeneration effect of cells and relapse the tumor.^171^

#### Inhibitory effects of mesenchymal stromal/stem cells on tumor growth

**Figure 3:**
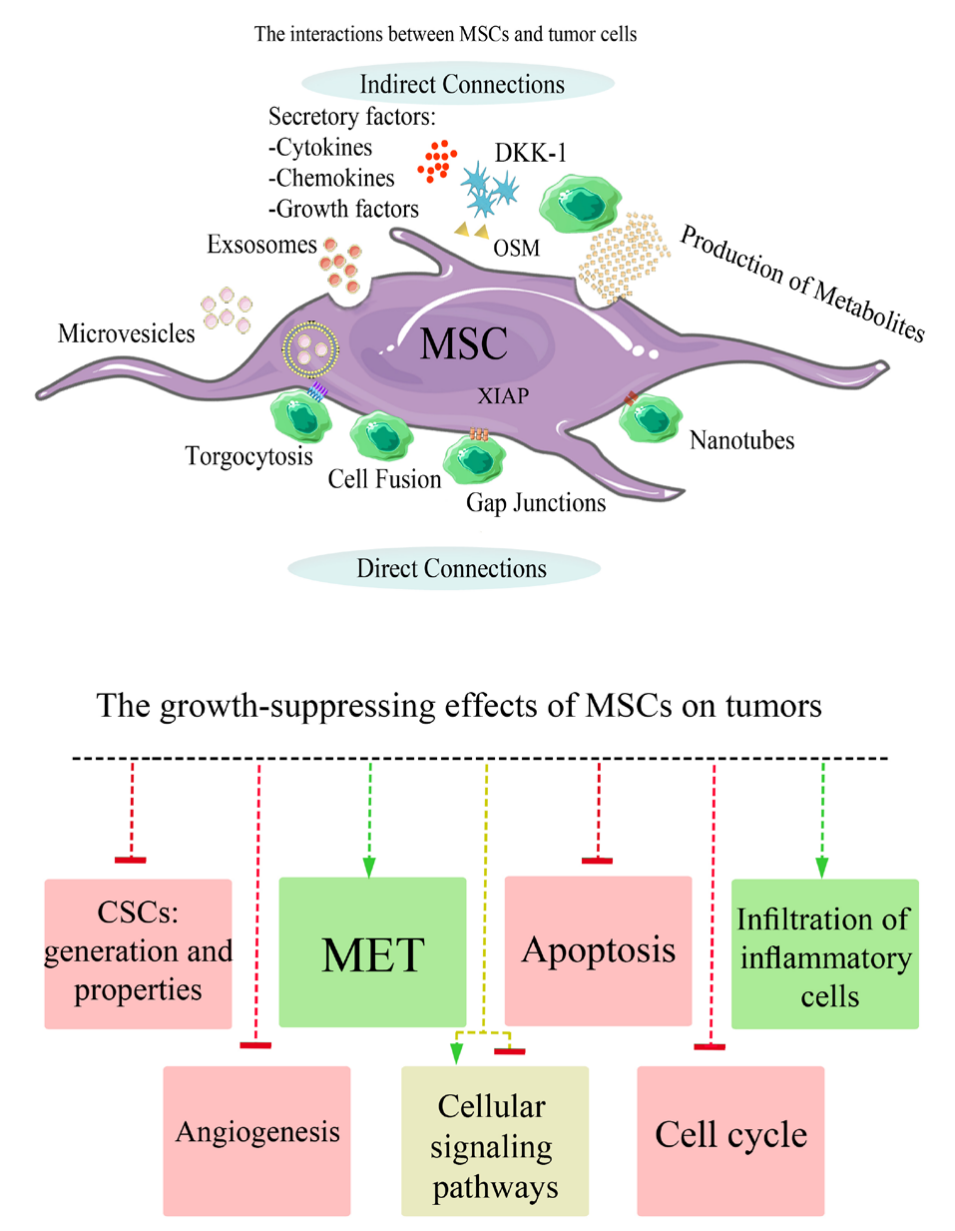
Different types of communication between MSCs and tumor cells and several different mechanisms adopted by MSCs to suppress tumor growth and progression through direct and indirect pathways. Some secreted factors such as MiR-122, IL-15, MiR-16, and OSM released by MSCs are capable of indirectly suppressing tumor progression through varying mechanisms. MET, mesenchymal-to-epithelial transition; XIAP, X chromosome-linked Inhibitor of Apoptosis Protein; DKK-1, Dickkopf-related protein 1; MSCs, mesenchymal stem cells.

Although a large number of results have shown that MSCs can significantly support tumor growth, progression, and metastasis some of the observations suggest that these cells can exert multiple suppressive effects on tumor development (Figure 3; Table 1 and 2).^23,172^

**Table 1:** Anticancer activities of the miRNAs which are detected in mesenchymal stem cell-derived extra cellular vesicles

**Types of cancer**	**Donors of extracellular vesicles-derived miRNAs**	**MicroRNAs**	**Target cells**	**Pathways which are involved in suppressive effects**	**Therapeutic effects**	**References**
Mouse breast cancer	MSC-derived exosomes	MiR- 16	Mouse mammary tumor cell line 4T1	Inhibition of angiogenesis through downregulation of VEGF	Inhibition of tumor growth	^178^
HCC	hAT -MSC-derived exosomes	MiR-122	HepG2^a^	-Reduction in ADAM1^b^, IGF-1R^c^, and CCNG1^d^ expression levels –Apoptosis induction -Cell cycle blocking	Sensitization of malignant cells to chemotherapy	^179^
-HCC -lymph Oma -Gliobla stoma	- HLSCs^e^ - MicroRNAs	MiR-31, MiR-451, MiR-223, MiR-122, MiR-125b, MiR-24, MiR-214	HepG2	-Reduction of tumor cells proliferation -Proapoptotic activities of: MiR-451, MiR-31, MiR-125b, MiR-24, MiR-223 which induces resistance to chemotherapy mediated by MDR1/P-gp^f^	-Inhibition of tumor growth -Reduction in survival of tumor cells	^180^
Breast cancer	hBMMSC-EVs^g^	MiR-23b	Bone marrow –derived metastatic human breast cancer cell line BM2	-Inhibition of MARCKS^k^ promoter which is involved in cell-cycle progression -The prevention of cancer cell motility -The inhibition of cancer cell proliferation	-Inhibition of tumor growth -A decrease in stem cell-like surface markers -Inhibition of invasion -An increase in resistance to chemotherapy	^181^
Glioma	MSCs	MiR-145 and MiR-124	-Human glioma cell lines A172 and U87 - GSCs^l^	Downregulation of SCP-1^m^ and SOX2^n^	A decrease in glioma cell migration and self-renewal of GSLCs^o^	^182^
Osteosarcoma	hBM-MSCs	MiR-143	Human osteosarcoma cell line 143B	-The delivery of MiR-143 to target tumor cells - The reduction of osteosarcoma cell migration	Reduction of metastasis	^183^
Glioma	MSC-EVS: MiR-146	MiR-146b	Human glioma cells	The binding of MiR-146b to EGFR^p^ mRNA and the inhibition of EGFR expression	Reduction of human glioma xenograft growth, invasion, and migration	^184^
MM^q^	BM-MSCs	micro RNA	Human multiple myeloma cell lines U266 and ARP-1	Inhibition of eIF1^r^ and eIF4G1^s^ (Involvement of transcription factors such as: NF-kB^t^, c-Myc^u^, CCND1^v^, HIF1α^w^, and Smad5^x^) -Inhibition of autophagy and tumor cell proliferation	Inhibition of tumor cells	^185^
-HCC -Kaposi's sarcoma -ovarian cancer	MSCs	MSC-EVs	-HepG2 -Kaposi’s sarcoma cell lines -Human ovarian carcinoma cell line SKOV-3	-Go/G1 cell cycle arrest -Apoptosis induction -Necrosis in SKOV3 cells	Inhibition of tumor growth	^186^
B-CLL^y^	MSCs	MiR-15/-16 family (MiR 15a/MiR-16-1)	Targeting of BCL-2, MCL1^z^, CCND1 gene -Anti-angiogenesis activities through Targeting VEGF-A^aa^ and Akt3^bb^	-Inhibition of tumor growth and progression	Under investigation	^187-191^
-pancre atic cancer -Breast cancer -Prostate cancer - Melanoma	MSCs	MiR-34	-Human melanoma cell line UACC-62 - p53 mutant pancreatic cancer cell line MIA PaCa-2 -Mutant p53 derived from metastasis of pancreatic cancer via cell-autonomous PDGFR Beta^cc^ signaling pathway - Human pancreatic cancer cell line BxPC3 -Human breast carcinoma cell line MDA-MB-231	-Re-expression of CD44+ in prostate cancer -Inhibition of clonogenic tumor cell growth and invasion -Apoptosis induction -Blockage of cell cycle in G1 and G2/M phases - Silencing NOTCH signaling pathway causes the downregulation of BCL-2 -Suppression of cell proliferation	-Reduction of tumor growth -Sensitivity to chemotherapy and radiation -Reduction in survival of CSCs^dd^ - There is probably no effect on melanoma	^192^
Prostate cancer	hBMMSCs	Let-7 MicroRNA	Cancer-associated MSCs which is co-cultured with human prostate cancer cell line PC3	Regulation of IL-6 expression and NF-kB in MSCs	There is probably an increase in Let-7 expression that cause the inhibition of tumor growth	^193^

^a^ HepG2, hepatocellular carcinoma cell line; ^b^ ADAM1, A disintegrin and A metalloprotease domain 1; ^c^ IGF-1R, the human type 1 insulin-like growth factor receptor; ^d^ CCNG1, cyclinG1; ^e^ HLSCs, human liver stem cells;^f^ MDR1, multidrug resistance protein 1 or P**-**glycoprotein; ^g^ hBMMSC-EVs, human bone marrow mesenchymal stemstromal cells derived extracellular vesicles ^h^ MARCKS, the myristoylated alanine-rich C-kinase substrate; ^l^ GSCs, human glioma stem cells; ^m^ SCP-1, the human small c-terminal domain phosphatase 1; ^n^ SOX2, SRY (sex determining region Y)-box 2; ^o^ GSLCs, human glioma stem-like cells; ^p^ EGFR, theepidermal growth factor receptor; ^q^ MM, multiple myeloma; ^r^ eIF1, eukaryotic translation initiation factor 1; ^s^ eIF4G1, eukaryotic translation initiation factor 4G1; ^t^ NF-kB, nuclear factor kappa beta; ^u^ c-Myc, cellular myelocytomatosis oncogene; ^v^ CCND1, Cyclin D1 gene; ^w^ HIF1α, hypoxia-inducible factor 1-alpha; ^x^ Smad5, mothers against decapentaplegic homolog 5 of the Drosophila gene; ^y^ B-CLL, B-cell chronic lymphocytic leukemia; ^z^ MCL1, myeloid cell leukemia sequence 1; ^aa^ VEGF-A, vascular endothelial growth factor A; ^bb^ Akt3, Akt serinethreonine kinase 3; ^cc^ PDGFR Beta,platelet derived growth factor receptor beta; ^dd^ CSCs, cancer stem cells.

**Table 2:** Anti-tumor or anti-metastatic activity of MSC-secreted factors

**Types of cancer**	**MSC donors and MSC products**	**The effective mediators**	**The target cells**	**The pathways and inflammatorymediators involved in the exertion of suppressive effects**	**The therapeutic effects**	**References**
HCC	hf MSC-CM^a^	IGF^b^	HCC cell lines	A decrease in IGF-1R^c^ activation and involvement of PI3K/Akt pathway; Cell cycle arrest	Inhibition of tumor growth and progression	^194^
Rat breast cancer	PMSCs^d^ BMMSC-CM	DKK-1^e^	Rat mammary tumor cell line	The blockage of Wnt/β-catenin signaling pathway	Inhibition of tumor cell growth, migration and invasion	^195^
Pancreatic tumor	hUCB-MSCs^f^	IL-15	Murine pancreatic adenocarcinoma cell line Pan02 or Panc02	Tumor cell apoptosis; Immunomodulatory activity affected by accumulation of NK, and T- cell, the promotion of T-cell immune memory responses	Inhibition of tumor growth	^196^
LAC^g^, Melanoma	MSCs or MSC-CM	OSM^h^	LAC cell line	Down regulation of STAT1^i^ through inhibition of Nanog and Slug expressions; Cell- cycle inhibition; Enhancement of MET^j^ process	Inhibition of tumor cell growth, invasion, and migration	^197^
Bladder tumor	hWJ-MSCs^k^	Unclear	Human bladder cancer cell line T24	Down regulation of Akt protein kinase; Phosphorylation and upregulation of cleaved caspase-3; Antiploriferative and proapoptotic effect; Cell cycle arrest	Inhibition of tumor growth	^198^
Breast cancer	hUC-MSCs^l^	IL-18	Human breast carcinoma cell line MCF-7	Alteration in cell cycle	Inhibition of tumor cell growth, invasion and migration	^199^
HCC	hBM-MSCs^m^	IFN-β^n^	HepG2- and Huh7-based human hepatoma cell lines	An increase in p21 and p27 expression; The decrease in cyclin D1 expression lead to cell cycle modification; A decrease in RB^o^ phosphorylation, suppression of Akt expression; Stimulation of FOXO3^p^ activity	Inhibition of tumor growth	^200^
Breast cancer	hAT-MSCs^q^, hATMSC-CM	IFN-β	Human breast carcinoma cell line MCF-7	The exertion of cytotoxic effects on breast cancer cells via STAT1 activation	Inhibition of tumor growth	^201^
Fibrosarcoma	MSCs	iNOS^r^	Fibrosarcoma cell line	Generation of NO or other cytotoxic agents and intermediate molecules	Inhibition of tumor growth or a significant tumor growth delay	^202^
Human mesothelioma, lung cancer, breast cancer, sarcomas, renal cancer, osteosarcoma, rhabdomyo sarcoma, ewing's sarcoma etc	MSCs	TRAIL^s^	-Human mesothelioma cell lines NCI-H2052,H2795, H2804, H2731, H2810,H2452, and H2869; Non small cell lung cancer cell lines NCI-H727, NCI-H460, A549, NCI-H23,and PC-9; Colon cancer cell lines COLO-205, RKO, and HT-29; Renal carcinoma cell lines RCC10 and HA7-RCC; Human oral squamous carcinomacell line H357	Apoptosis induction; The attenuation ofinflammatory TME; Inhibition­ of angiogenesis	-Inhibition of tumor growth and progression	^203-206^

^a^ hfMSC-CM, human fetal mesenchymal stem cell-derived conditioned media; ^b^ IGFs, insulin like growth factors; ^c^ IGF1R, insulin like growth factor 1 receptor; ^d^ PMSCs, rib perichondrium mesenchymal stem stromal cells; ^e^ DKK-1 Dickkopf-related protein 1; ^f^ hUCB-MSCs, human umbilical cord blood-derived mesenchymal stem stromal cells; Nk, natural killer; ^g^ LAC, Lung adenocarcinoma; ^h^ OSM, oncostatin M; ^i^ STAT1, signal transducer and activator of transcription 1; ^j^ MET, mesenchymal-epithelial transition; ^k^ hWJ-MSCs, Human Whartson’s Jelly Derived Mesenchymal StemStromal cells; ^l^ hUC-MSCs, Human Umbilical Cord Blood-Derived Mesenchymal StemStromal cells; ^m^ hBM-MSCs, Human Bone Marrow -Derived Mesenchymal Stem Cells; ^n^ IFN-β, interferon beta; ^o^ RB, retinoblastoma; ^p^ FOXO3, Human forkhead box protein O3; ^q^ hAT-MSCs, human adipose tissue-derived mesenchymal stem stromal cells; ^r^ iNOS, inducible nitric oxide synthase; ^s^ trail, tumor necrosis factor-related apoptosis-inducing ligand

The suppressive effects of MSCs have been reported in several human tumor types such as liver cancers, leukaemia, Kaposi’s sarcoma, pancreatic cancer, and melanoma.Anti-tumor property of MSCs function through exosomes which is derived from cells and also these cells could be used as an anti-tumor reagent carrier through the ability of migration to tumor site and integrate into tumor (Table 1).^26,28,120,173-177^

#### The induction of apoptosis in tumor cells

MSCs can induce apoptosis in different tumor cells through direct or indirect pathways (Figure 3).^173,203^ For instance, MSC- conditioned media (MSC-CM) can induce both apoptosis and autophagy in tumor cells^207^ (Tables 1 and 2). Sandra et al^208^ provided compelling evidence that MSC-derived secretome could reduce cell proliferation of the human cervical cancer HeLa cell line in a time and concentration-dependent manner by the induction of tumor cell apoptosis.^208^ Moreover, human umbilical cord-derived mesenchymal stromal/stem cells (hUC-MSCs) display pro-apoptotic and anti-proliferative effects on human prostatic carcinoma cell line PC3 through direct and indirect communication in an in vitro co-culture system that accompanied by activation of c-Jun N-terminal kinase (JNK) and inhibition of phosphoinositide 3-kinase/protein kinase B, also known as Akt signaling pathways.^209^ It has reported that the modification of MSCs with IFN-γ causes a high-level production of functional IFN-γ that can result in a high-level expression of tumor necrosis factor-related apoptosis-inducing ligand (TRAIL)/Apo-2 ligand (Apo2L) and through which MSCs can exhibit pro-apoptotic effects on tumor cells.^210^ Furthermore, human umbilical cord blood-derived stromal/stem cells (hUCB-MSCs) induce apoptosis in U257 glioblastoma cell line through depletion of X-chromosome linked inhibitor of apoptosis protein (XIAP) which in turn lead to the activation of caspase-9/caspase-3 pathway in tumor cell lines.^211^

#### Inhibition of cell cycle progression

The previous studies have elucidated that MSCs isolated from various sources including breast tissue, human palatine tonsils, and adipose tissue exert the inhibitory effects on tumor cell proliferation by inducing cell-cycle arrest in G0/G1 phase (Figure 3; Table 1 and 2).^175,212^ In addition, *ex vivo* expansion of human amniotic mesenchymal tissue cells (hAMTCs) through co-culturing with different types of human tumor cell lines have shown anti-proliferative effects on tumor cells. The microarray data have exhibited a significant decrease in expression of cyclin D1, cyclin E1, cyclin H, cyclin-dependent kinase (CDK) inhibitor p15^INK4b^, and CDK inhibitor p21^Waf1/Cip1^ which is along with an increase in expression of retinoblastoma (RB) gene that finally leads to G0/G1 cell cycle arrest in tumor cells. Previous studies have also revealed that RB1 (p107) which normally acts as a transcriptional repressor markedly downregulates during G0/G1 cell cycle arrest.^213^

#### Inhibition of specific cell signaling pathways

Multiple signal transduction pathways have been expected to be involved in tumor suppressive effects adopted by MSCs. The signaling pathways can be affected either directly or indirectly by MSCs. For instance, the PI3K/Akt signaling pathway has an important role in biological functions of tumor cells such as proliferation, apoptosis, differentiation, and oncogenic activities.^90,214^ Experiments have been shown that hUC-MSCs exert apoptotic and anti-proliferative effects on human prostatic carcinoma cell line PC3 via activation of JNK and inhibition of PI3K/Akt signaling pathways, in indirect and direct co-culture system.^209^ Strikingly, the NF-kB family which is well known as a critical transcription factor related to the inflammation plays a critical role in tumor progression. It has already been shown that MSCs have also the ability to inhibit NF-kB in tumor cells (Table 1).^214^ Furthermore, MSCs can inhibit the proliferation-related signaling pathways through paracrine actions (Figure 3). For instance, MSCs can produce and release Dickkopf-related protein 1 (Dkk-1) which in turn inhibit the expression of Wnt downstream targets and/or effectors such as BCL-2, cellular myelocytomatosis oncogene (c-Myc), β-catenin, BAX, and survivin in tumor cells (Table 2).^177,215^

#### Inhibition of tumor angiogenesis

There is considerable observational evidence that suggests MSCs have the ability to inhibit tumor angiogenesis and development. The anti-angiogenic effects of MSCs can be induced in a concentration-dependent manner in several tumor types.^215^ An *in vitro* co-culture of BM-MSCs with melanoma cell lines has been shown that MSCs seem to produce locally cytotoxic molecules which are responsible for the blockage of capillary network formation, inhibition of tumor angiogenesis, and finally suppression of tumor growth and progression (Figure 3 and Table 1).^215,216^ It has also revealed that ROS generated by MSCs act like cytotoxic agents. Similarly, the oxidative stress directly affects gene expression profiles of vascular endothelial cells (VECs) that are accompanied by down-regulation of angiogenic cascade such as PDGF, platelet derived growth factor receptor (PDGFR), vascular endothelial-cadherin (VEC/VE-cadherin), and β-catenin. The process ultimately leads to the suppression of VEGF expression in a concentration-dependent fashion. It is also noteworthy that the process happens through direct communication between MSCs and VECs that finally leads to the co-location of VE-cadherin/β-catenin in the plasma membrane of endothelial cells.^215-217^

MSCs can also prevent formation of new blood vessels and tumor angiogenesis by the induction of apoptosis in vascular endothelial cells.^217^

#### Induction of mesenchymal-epithelial transition

Emerging evidences suggest that the EMT is a critical and complex phenomenon that determines tumor invasiveness and the metastatic potentials of different types of tumors. The highly conserved molecular machinery which is responsible for controlling EMT can shift the process in the direction previously intended known as mesenchymal-epithelial transition (MET) (Figure 3).^218-220^ Previous studies have been revealed that co-culture of lung adenocarcinoma (LAC) cell lines with MSCs leads to the suppression of tumor development, invasion, and tumor cell migration. Both MSCs and MSC-CM exert suppressive effects on several cancer cell lines through down-regulation of EMT-related markers and enhancement of MET pathway (Table 2). Moreover, it has been suggested that oncostatin M (OSM) as a paracrine factor has a decisive role in stimulating of MET process through the activation of signal transducer and activator of transcription 1 (STAT1) pathway.^219^

#### Polarization of MSCs into a pro-inflammatory MSC1 or anti-tumor phenotype

Over the past decade the number of clinical trials involving MSCs has steadily increased, resulting in MSCs being the most commonly used cell type in tissue engineering, regenerative medicine and damage repair. These adult multipotent stem cells are massively proliferative and hold abilities to differentiate into wide variety of cell types. MSCs have been derived from various organs of healthy human subjects comprise a heterogeneous mixture of progenitor cells which are considered as suitable candidates for cell-based cancer therapies with different abilities. Substantial clinical trials confirmed that MSCs are frequently utilized cell types for regenerative medicine and tissue engineering. These cells express a diverse array of TLRs including TLRs1, 2, 3, 4, 5, 6, and 9.^221-223^ TLRs can recognize exogenous and endogenous danger signals and have profound effects on proliferation, differentiation, migration, and survival of MSCs.^221^ TLRs have been identified in different MSCs include BM-MSCs, human adipose-derived mesenchymal stromal/stem cells (hA-MSCs), human umbilical cord blood Wharton’s jelly-derived mesenchymal stromal/stem cells (hUCBWJ-MSCs), hUC-MSCs, human dental follicle-derived mesenchymal stem/stromal cells (hDF-MSCs), and human dental pulp-derived mesenchymal stem/stromal cells (hDP-MSCs).^224-231^

Toll -like receptors may also significantly affect the interaction between immune cells and MSCs.^226-227,230^ It is essential to note that the expression and function of TLRs in MSCs can be modulated under certain physiological conditions. For instance, an inflammatory milieu or exposure to bacterial components may determine the expression and function of TLRs in MSCs that lead to priming of specific TLRs and the alteration of immunomodulatory effects of MSCs.^232-234^ The expression pattern of TLRs in MSCs always coordinates with their origins.^233^

According to a new paradigm MSCs can be deeply polarized into two distinct phenotypes of MSCs named MSC1 and MSC 2 (MSC2) which the resulting phenotypes are based on their TLR- priming that exhibit opposite effects on tumor development.^39,235,236^ The new paradigm suggests that TLR4-primed MSCs or MSC1 exert mainly pro-inflammatory or anti-tumor activities.^235,237^ While TLR3-primed MSCs or MSC2 display mostly anti-inflammatory and pro-tumor properties.^235^ Experimental evidence suggests that co-culture of MSC1 with different types of various cancer cell lines leads to a decrease in the number of colony-forming units derived from tumor cells and the reduction of three-dimensional (3D) tumor spheroid invasion assay. Meanwhile, a paradoxical result obtained using the conventionally prepared MSCs or MSC2 when co-cultured with various cancer cell lines.^235^

Romieu-Mourez et al^228^ have demonstrated that the stimulation of distinct TLRs in mouse-derived MSCs caused pro-inflammatory cytokines production including IL-8, IL-1, IL-6, and CCL5 by MSCs. They also argued that co-administration of TLR-primed MSCs (which are activated by IFN-γ) and extracellular matrix components (for instance, Matrigel Matrix) led to the generation of a local inflammatory site. Then the created site of local inflammation could attract and regulate the immune cells. Briefly, main changes occurred in this phenomenon including recruitment of neutrophils, secretion of different profiles of cytokines , alterations in the differentiation capability of MSCs, deposition of ECM; and also expression of the TGF-β, IDO, PGE2, and jagged-2 by MSCs.^228,236^

More studies on MSC polarization may provide a convenient way to prevent the adverse effects of unmodified MSCs on tumor progression.

## Conclusion

In the recent decade, there exists an increasing trend in the use of MSCs as an attractive therapeutic option in case of cancer therapy, regenerative medicine, and various human diseases. MSCs have unique characteristics such as the ability to migrate toward the primary tumor and metastatic sites, anti-tumor properties, and homing to be crucial determinants of tumor cell fate. For instance, MSCs in its unmodified or native form are being extensively studied and hold great promise for utilization in stem cell-based therapies for cancer because of long time survival of exogenous MSCs in tumor mass. However, more findings imply that MSCs when confronted with tumors could exert dual and opposite effects on tumor growth and progression which depend on different conditions and factors. These divergent effects of MSCs in tumors can be considered as a major obstacle to successful stem cell-based therapies for cancer. It is also apparent that MSCs exert their contradictory effects on tumor growth and progression through different mechanisms. Hence, expanding the knowledge of these mechanisms involved in the control of tumor growth and their activation/deactivation process can result in more effective clinical outcomes. Prevention of the adverse effects of stem celltransplantation and the evolution of phenotypes which have anti-tumor effects can be one of the main goals of the research on the application of intact MSCs for the treatment of cancer. As the ability of homing phenomena and extended survival in tumor site, these cells could be genetically engineered for anti-cancer drug and reagent vehicle for therapeutic purposes. It should be noted that supportive effects of MSCs are predominant compared with their inhibitory effects on tumor development. Also, various conditions and factors can play a significant role in exhibiting of bidirectional effects of MSCs on tumor development.

## Ethical Issues

Not applicable.

## Conflict of Interest

Authors declare no conflict of interest in this study.

## Acknowledgments

The authors would like to express their deepest gratitude and appreciation to the Department of Molecular Medicine, Faculty of Advanced Medical Sciences, Tabriz University of Medical Sciences, and the Drug Applied Research Center at Tabriz University of Medical Sciences, Tabriz-Iran whose assistance proved to be a milestone in the accomplishment of this paper.
